# Encapsulation of Alpha-1 antitrypsin in PLGA nanoparticles: In Vitro characterization as an effective aerosol formulation in pulmonary diseases

**DOI:** 10.1186/1477-3155-10-20

**Published:** 2012-05-20

**Authors:** Nazanin Pirooznia, Sadegh Hasannia, Abbas Sahebghadam Lotfi, Mostafa Ghanei

**Affiliations:** 1 Department of Biology, Faculty of Sciences, University of Guilan, Rasht, IR, Iran; 2 Faculty of Biological Sciences, Tarbiat Modares University, Jalal Ale Ahmad Highway, P.O.Box: 14115-111, Tehran, IR, Iran; 3 National Institute of Genetic Engineering and Biotechnology, Tehran, IR, Iran; 4 Department of Clinical Biochemistry, Faculty of Medical Sciences, Tarbiat Modares University, Tehran, IR, Iran; 5 Research Center for Chemical Injuries, Baqiyatollah University of Medical Sciences, Tehran, IR, Iran

**Keywords:** Cytotoxicity, DSC (differential scanning calorimetry), FTIR, Nanoparticle, Sustained drug release, XRD (x-ray diffraction)

## Abstract

**Background:**

Alpha 1- antitrypsin (α1AT) belongs to the superfamily of serpins and inhibits different proteases. α1AT protects the lung from cellular inflammatory enzymes. In the absence of α1AT, the degradation of lung tissue results to pulmonary complications. The pulmonary route is a potent noninvasive route for systemic and local delivery. The aerosolized α1AT not only affects locally its main site of action but also avoids remaining in circulation for a long period of time in peripheral blood. Poly (D, L lactide-co glycolide) (PLGA) is a biodegradable and biocompatible polymer approved for sustained controlled release of peptides and proteins. The aim of this work was to prepare a wide range of particle size as a carrier of protein-loaded nanoparticles to deposit in different parts of the respiratory system especially in the deep lung. Various lactide to glycolide ratio of the copolymer was used to obtain different release profile of the drug which covers extended and rapid drug release in one formulation.

**Results:**

Nonaqueous and double emulsion techniques were applied for the synthesis of nanoparticles. Nanoparticles were characterized in terms of surface morphology, size distribution, powder X-ray diffraction (XRD), encapsulation efficiency, *in vitro* drug release, FTIR spectroscopy and differential scanning calorimetry (DSC). To evaluate the nanoparticles cytotoxicity, cell cytotoxicity test was carried out on the Cor L105 human epithelial lung cancer cell line.

Nanoparticles were spherical with an average size in the range of 100 nm to 1μ. The encapsulation efficiency was found to be higher when the double emulsion technique was applied. XRD and DSC results indicated that α1AT encapsulated in the nanoparticles existed in an amorphous or disordered-crystalline status in the polymer matrix. The lactic acid to glycolic acid ratio affects the release profile of α1AT. Hence, PLGA with a 50:50 ratios exhibited the ability to release %60 of the drug within 8, but the polymer with a ratio of 75:25 had a continuous and longer release profile. Cytotoxicity studies showed that nanoparticles do not affect cell growth and were not toxic to cells.

**Conclusion:**

In summary, α1AT-loaded nanoparticles may be considered as a novel formulation for efficient treatment of many pulmonary diseases.

## Background

Alpha 1- antitrypsin (α1AT) is a 54 kDa glycoprotein which belongs to the superfamily of serpin. The protein inhibits serine protease and a broad group of other proteases. It protects the lungs from cellular inflammatory enzymes, especially elastase, therefore it is known as the human neutrophil elastase inhibitor [1,2]. In the absence of α1AT, the neutrophil elastase released by lung macrophages, is not inhibited, thus leading to elastin breakdown and the loss of lung elasticity. This causes degradation of the lung tissue resulting in pulmonary complications, such as emphysema or chronic obstructive pulmonary disease COPD in adults [3,4]. Impaired α1AT secretion in liver and accumulation in liver cells also causes cirrhosis in infants [5,6]. Relation between α1AT deficiency and various diseases including asthma, rheumatoid arthritis, anterior uveitis and systemic lupus erythematosus has also been reported [7-9]. α1AT is not only an anti-inflammation protein but also an immune system regulator which regulates lymphocyte proliferation and cytotoxicity, mediates monocyte and neutrophil functions. Besides antiapoptotic function in the lung epithelial cells *in vitro*[10], has broad anti-inflammatory effects in humans [11]. It has been shown that protease-antiprotease imbalance is an important factor in the pathogenesis of COPD and other pulmonary diseases, such as bronchitis. In this process, an exogenous proteolytic enzyme leads to lung tissue breakdown (because human neutrophil elastase cannot be inhibited by α1AT) [12,13]. Furthermore, α1AT can act as a tumor suppressor by inhibiting the growth of breast cancer cells [14].

The treatment of cystic fibrosis (CF) patients with aerosolized α1AT has been shown to reduce sputum neutrophil numbers, IL-8 concentration and elastase function [15]. The effect of α1AT on IL-8 as a neutrophil chemoattractant and a neutrophil recruiter to the lung is also important because neutrophils are considered as the main cause of COPD pathogenesis and clinical effects of α1AT in COPD are obvious [16].

A recent study has shown that α1AT can slow down the loss of insulin producing cells in diabetic patients who are at the early stages of disease. This study, which is in phase II of clinical trials, demonstrates the effect of α1AT in preservation of β-cells, function to produce insulin [17,18].

The commercially available plasma derived product of α1AT is administered intravenously. Such an intravenous augmentation therapy has disadvantages, such as high costs, viral contaminations and immune reactions because of prolonged retention of the drug in circulation. The pulmonary route is an alternative, potent and noninvasive route for systemic and local delivery of macromolecules. The aerosolized α1AT not only affects locally the lung, its main site of action, but also avoid remaining and circulation for a long time in peripheral blood [19].

This route of administration provides large surface area, thin epithelial barrier, avoids first-pass metabolism and high blood flow. Many proteins and peptides have been delivered to the lung successfully in this way [20,21]. In fact spherical materials less than 10 μ in diameter can be inhaled. Particles less than 2.5 μ can reach the alveoli. Nanoparticles less than 100 nm are mainly deposited in the alveolar region. Nano-sized particles avoid macrophage pulmonary clearance and cross without active uptake [22].

The aerosolized α1AT is currently under study [8,23]. In the aerosol form, 25%-45% of aerosolized particle reaches the respiratory system, however in the intravenous infusion only 10%-15% of α1AT reaches to this site. The aerosolized α1AT not only affects its main site of action locally, but also avoids remaining in circulation for a long period of time in peripheral blood, thus avoiding immune response reactions because of long-term use of α1AT [20,21].

Nevertheless, for the purpose of drug delivery, PLGA (poly- D, L lactide-co glycolide) is the best candidate. PLGA-based nanotechnology has gained tremendous interest in medical applications such as sustained drug release, drug delivery, diagnostics and treatment [24]. Polylactide (PLA) and its copolymers that contain glycolide (PLGA) have been approved by the US food and drug administration (FDA) for the purpose of drug delivery. PLGA is an ideal choice for drug delivery because of its unique properties including biocompatibility, bioavailability and variable degradation kinetics, high drug-loading capability, stability and extended drug release over other carriers such as liposomes [25-28]. PLGA protects the encapsulated drug from enzymatic degradation and changes the pharmacokinetics of the drug. It provides a wide range of degradation rates, from months to years, depending upon its composition and molecular weight^27^.FDA has approved products using PLGA as carriers which include Nutropin Depot for growth deficiencies, Sandostatin LAR for acromegaly and Trelstar Depot for prostate cancer [29]. At present many other PLGA-based formulations are also at the pre-clinical stage.

The biodegradation rate of PLGA polymers is dependent on the lactide/glycolide, molecular weight, degree of crystallinity and the transition glass temperature (Tg) of the polymer [30]. The release profile of PLGA nanoparticles can be divided into 4 different phases: initial burst, induction period, slow release period and final release period [31]. Polymers containing a 50:50 ratios of lactic and glycolic acid have faster hydrolytic activities than those with other ratios of the monomers. PLGA nanoparticles can be used safely for oral, nasal, pulmonary, parenteral, transdermal and intra-ocular routes of administration [32]. The PLGA nanoparticles can be prepared by different techniques. The most common technique is the emulsification solvent evaporation technique because of its simplicity and high encapsulation efficiency. The single emulsion method is only suitable for hydrophobic drugs and leads to very poor encapsulation efficiency with regard to protein or peptide drugs. The oil-in-oil (o/o) emulsification technique which is known as the nonaqueous emulsion method is a new and efficient method for encapsulation of hydrophilic drugs. The double emulsion method is also suitable for encapsulating hydrophilic drugs with high efficiency [20,33,34].

In this work, we prepared α1AT-loaded nanoparticles with two different methods and studied the release properties of nanoparticles at different ratios of lactide/glycolide. The polydispersity of nanoparticles is an important factor in this work due to the lung's anatomy.

Finally the nanoparticles which were prepared by the double emulsion technique were mixed to obtain a 50:50 and 75:25 ratios of nanoparticles fabricated with PLGA. In this way the capabilities of the mixture was explored for the simultaneous prolonged and high initial burst release of the drug.

## Results and discussion

### Image analysis

**Figure 1:**
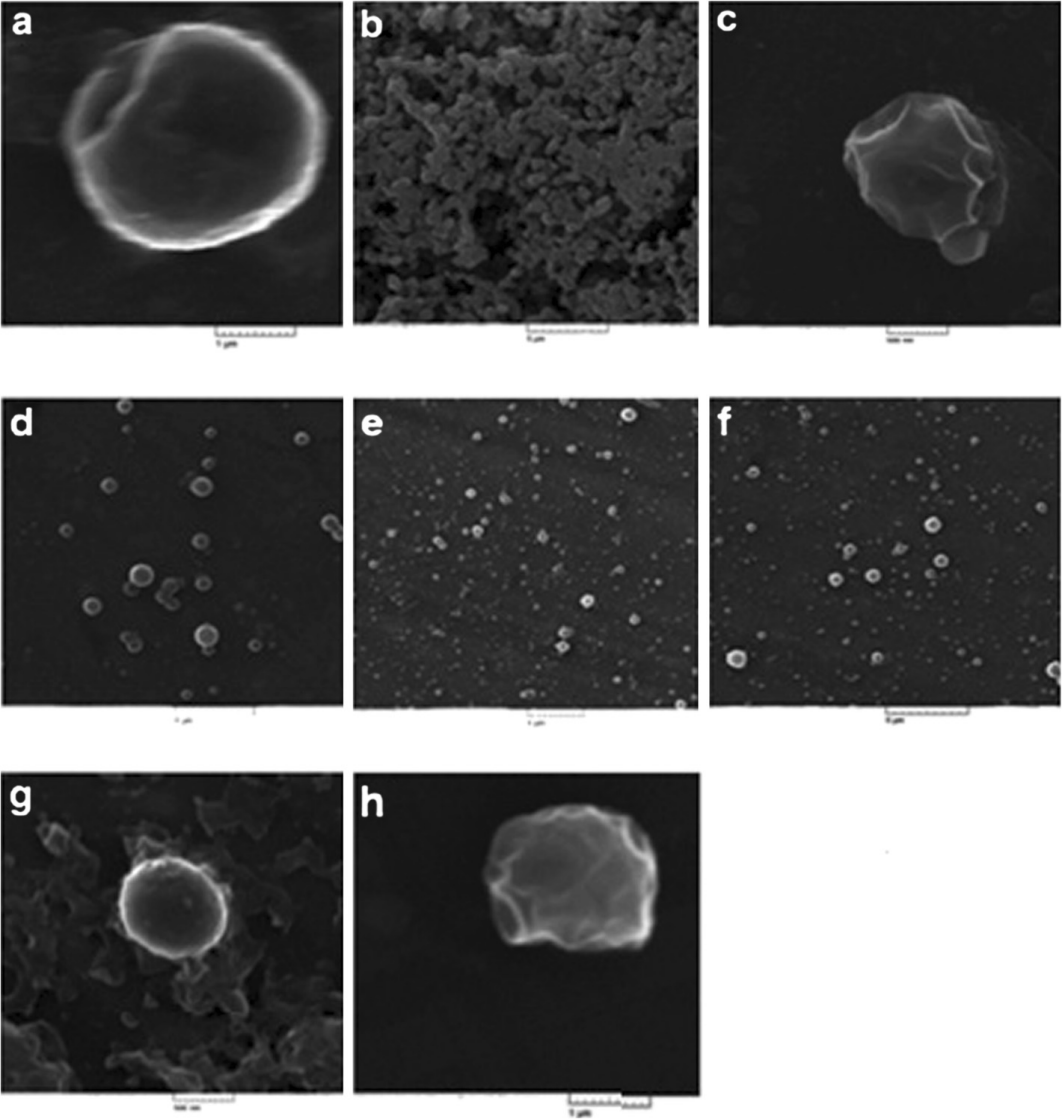
Typical SEM image of AAT-loaded (a, b, c, d, f, g and h) and unloaded nanoparticles (e).

The SME graphs revealed the particle size and surface morphology of protein-loaded PLGA nanoparticles with copolymer ratio of lactide: glycolide 50:50 and 75:25. The morphology of nanoparticles prepared by double and nonaqueous emulsion technique was spherical. Protein loaded-particles have smooth surface with few indents that can be because of α1AT incorporation. Results showed that particles have nano range size ~350 nm. Micrographs of freeze-dried nanoparticles without any stabilizer such as sorbitol showed that PLGA nanoparticles have the tendency to form aggregates; therefore in preparation process sorbitol 1% (w/v) was added in the final step before lyophilization. The initial and final particles mean size and distribution were identical before and after freeze-drying process (Figure 1).

### Particle size and distribution

**Figure 2:**
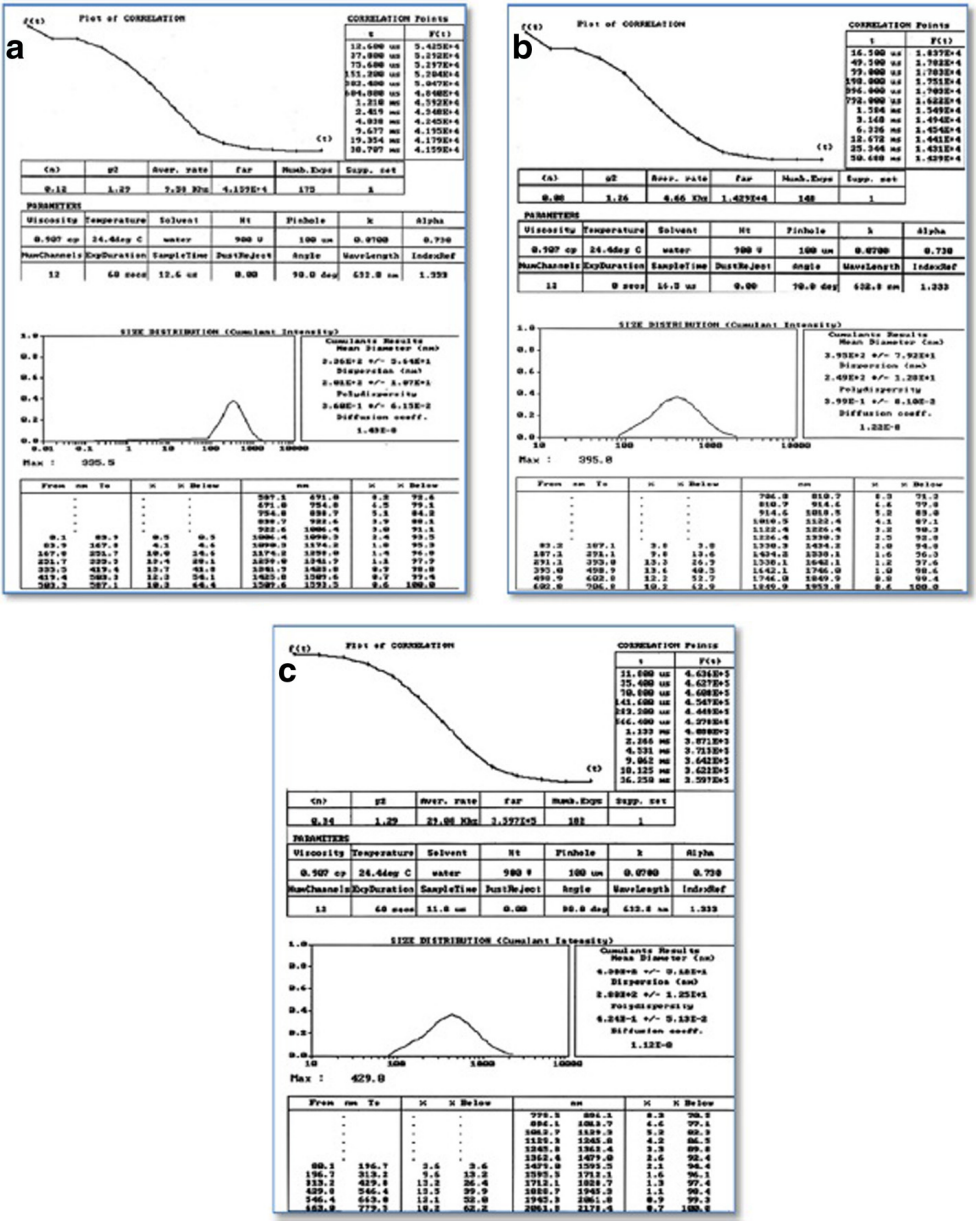
mean particle size of nanoparticle prepared with nonaqueous (a), double emulsion technique using PLGA 50:50 (b) and 75:25 (c).

The particle size distribution of prepared nanoparticle by nonaqueous and double emulsion techniques and different lactide: glycolide ratio is summarized in Figure 2. Preparation method affects particle size and distribution. Particles produced by nonaqueous emulsion technique have smaller size and wider size distribution. Nanoparticles obtained by double w/o/w technique have slightly bigger size and narrower distribution. Although the polymer ration has no effect on particle size and its distribution. α1AT-loaded PLGA (50:50 and 75:25) nanoparticles produced by double emulsion technique have similar size and particle distribution (Figure 2).

### The X-Ray diffraction (XRD)

**Figure 3:**
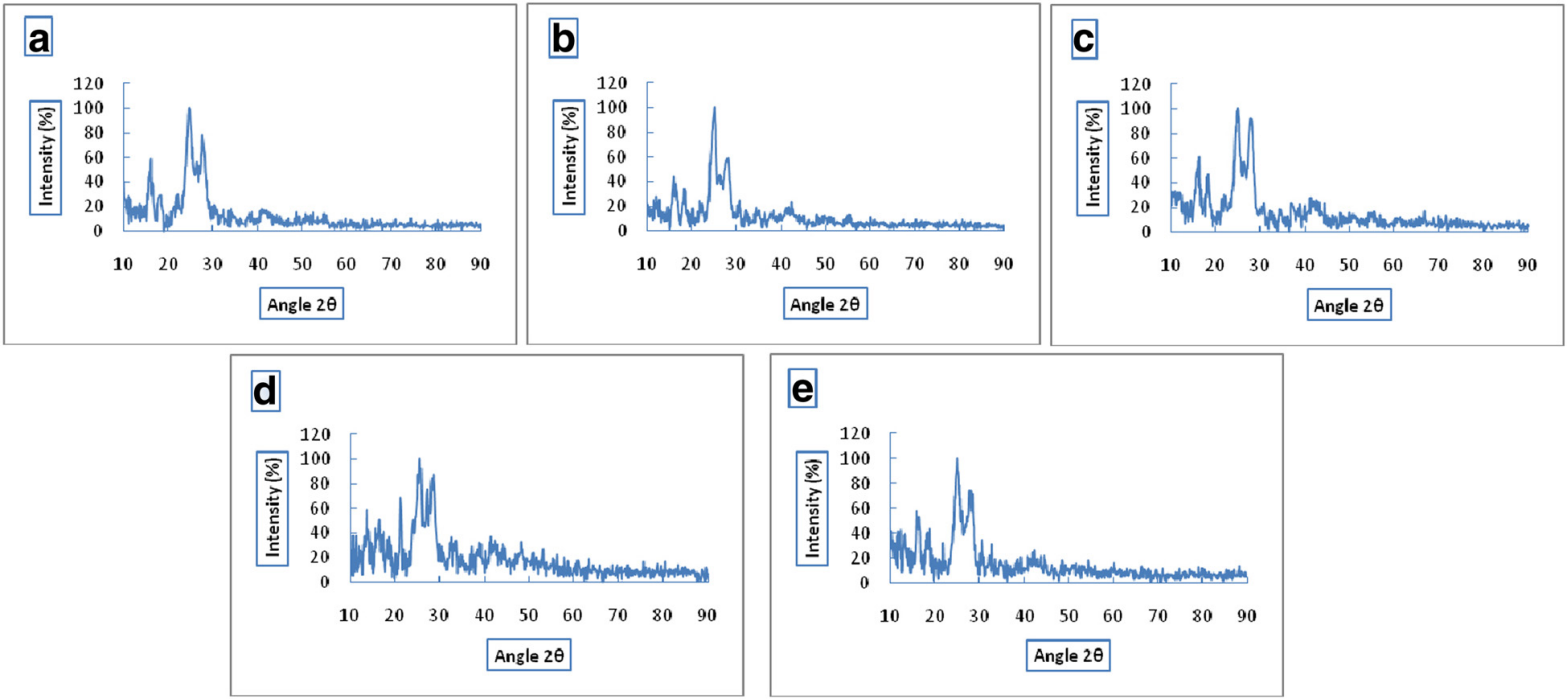
XRD spectra of PLGA 50:50 (a), PLGA 75:25 (b), AAT- loaded PLGA nanoparticle prepared using nonaqueous emulsion technique (c), AAT- loaded nanoparticle prepared using double emulsion technique containing PLGA 50:50 (d) and AAT- loaded nanoparticle prepared using double emulsion technique containing PLGA 75:25 (e).

The XRD results showed a peak between 3° and 5° (2) which correspond to the semi crystalline state for the α1AT protein. Results indicate that α1AT in nanoparticles has an amorphous or disordered-crystalline state which can possibly be due to the protein distribution in polymer matrix. No peak was observed for PLGA with different ratio which indicates that PLGA is an amorphous copolymer (Figure 3).

### Loading efficiency

The encapsulation yield was calculated by the following equation:

(1) $$\text{Encapsulation}\;\text{efficiency}\left(\%\right)=\frac{\text{The}\;\text{protein}-\text{loaded}\;\text{nanoparticle}\;\text{weight}-\text{protein}\;\text{concentration}\;\text{after}\;\text{hydrolysing}}{\text{The}\;\text{initail}\;\text{amount}\;\text{of}\;\text{protein}\;\text{added}\;\text{during}\;\text{synthesis}}\times 100$$

Protein concentration after hydrolyzing by fluorometric method and encapsulation yield was as shown in Table 1.

**Table 1:** Encapsulation efficiencies applying different PLGAs and preparation techniques

**Applied technique**	**Copolymer ration of PLGA**	**Protein concentration (mg/ml)**	**Encapsulation yield**
Double emulsion technique	75:25	0.39	% 92.2 ± 5.5
Double emulsion technique	50:50	0.27	% 94.6 ± 5.5
Nonaqueous emulsion technique	50:50	0.6	% 88 ± 5.5

As a result, the double emulsion technique for the preparation of nanoparticles leads to a higher entrapment efficiency of α1AT. Results showed that difference in encapsulation efficiency was not significant for PLGA 50:50 and 75:25, therefore the copolymer ratio does not affect the drug encapsulation efficiency.

### *In vitro* release

**Figure 4:**
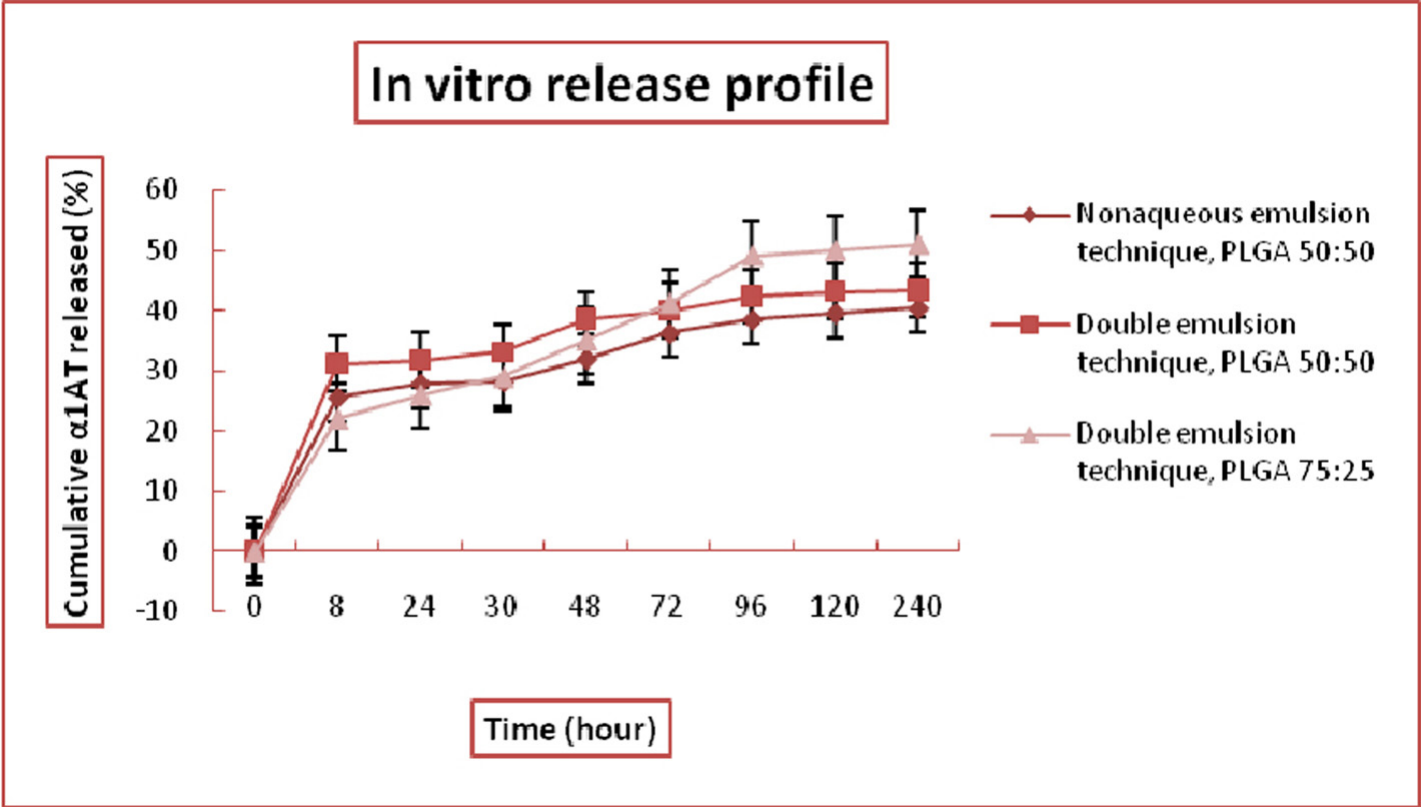
In vitro release profile of α1AT from PLGA nanoparticles with different ratio synthesized by nonaqueous and double emulsion technique.

The standard curve was generated using a series of dilutions ranging from 2.5 to 200 μg/ml of α1AT. The concentration of the released protein was determined using intrinsic fluorescence of aromatic amino acids. This technique is suitable for quantitating protein concentrations of less than 10μg/ml Fluorescence was measured using excitation/emission wavelengths of 280/332nm. The intensity of fluorescence correlates to the protein concentration (Figure 4 and Table 2).

**Table 2:** Fluorescence intensity of protein release at different time intervals from PLGA nanoparticles which reflect the protein concentration

**Technique Time**	**Nonaqueous emulsion PLGA 50:50**	**Double emulsion PLGA 50:50**	**Double emulsion PLGA 75:25**
**0**	0	0	0
**8**	7.69	9.33	6.65
**24**	8.38	9.54	7.81
**30**	8.64	9.94	8.69
**48**	9.61	11.58	10.54
**72**	10.9	11.99	12.38
**96**	11.58	12.74	14.74
**120**	11.89	12.95	15.02
**240**	12.12	13	15.31

### Protein polymer interaction study

**Figure 5:**
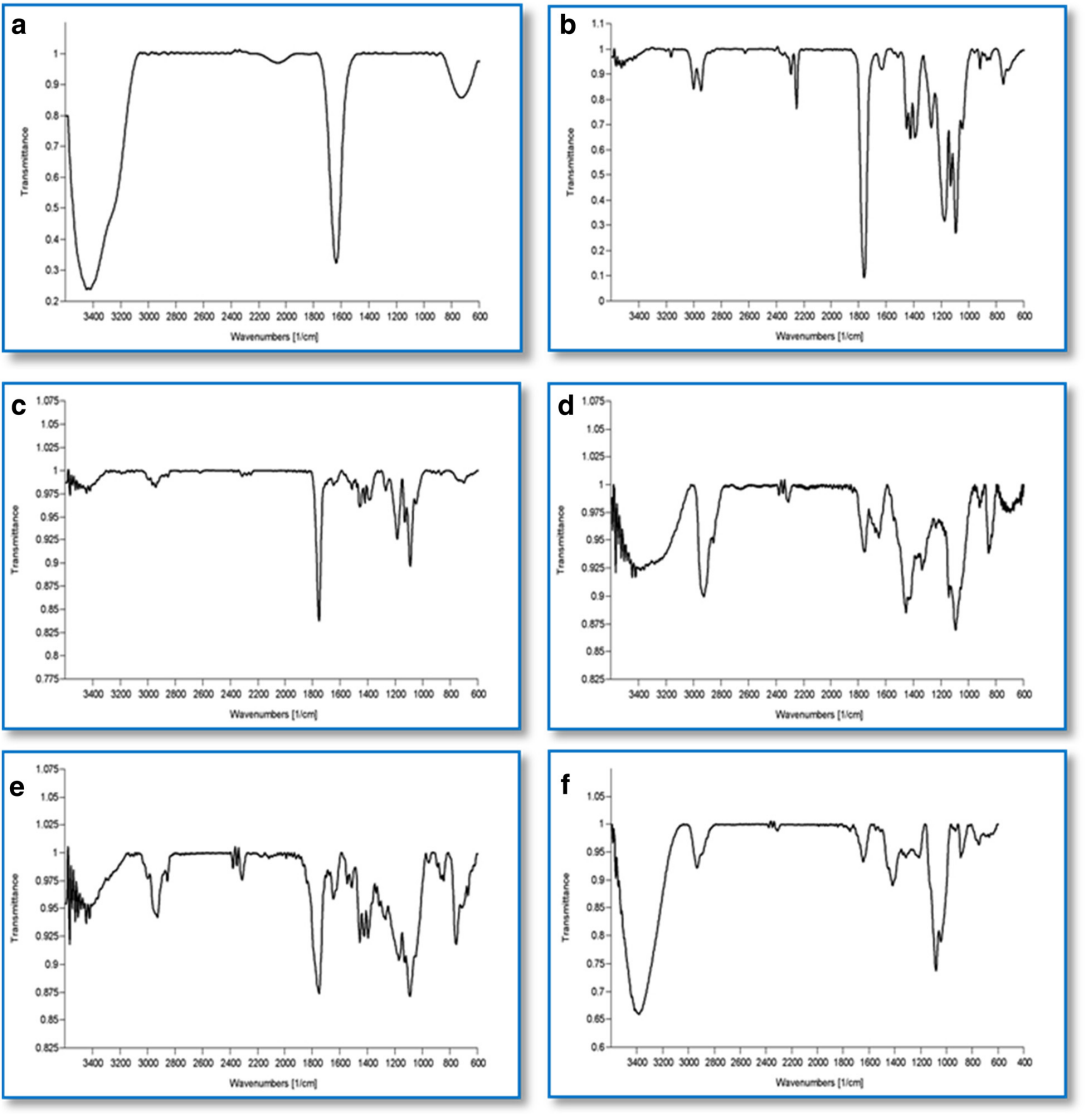
FTIR spectra of pure AAT (a), PLGA 50:50 (b), PLGA 75:25 (c), nonaqueous emulsion made α1AT-loaded PLGA (d), double emulsion made α1AT-loaded PLGA (50:50) and (75:25)(e and f, respectively).

The FTIR spectra of α1AT, PLGA 50:50 and 75:25, α1AT- loaded PLGA (50:50 and 75:25) show that there were no changes in the position of absorption peaks. The pure AAT sample (Figure 5a) showed the main peaks contributed by the functional groups of molecule such as carbonyl –C = O stretching (1650 cm^-1^), –OH stretching (3300-3500 cm^-1^), –CH twisting (900-1000 cm^-1^). The pure PLGA 50:50 (Figure 5b) and 75:25 samples (Figure 5c) showed peaks such as OH stretching (3200-3500 cm^-1^), –CH (2850-3000 cm^-1^), carbonyl –C = O stretching (1700-1850 cm^-1^) and C–O stretching (1050-1250 cm^-1^). AAT-loaded nanoparticles using nonaqueous emulsion (Figure 5d) and double emulsion technique with PLGA 50:50 and 75:25 (Figure 5e and f) showed peaks resulting from simple superposition of their separated components in the infrared spectra. Spectral analysis indicated that the specific functional groups of polymeric material in the nanoparticles surface have almost the same chemical characteristics of the pure polymer. The study suggests that molecular interactions that could alter the chemical structure of the drug did not occur. Therefore, no chemical interaction between functional group of protein and polymer exist (Figure 5).

### Differential Scanning Calorimetry measurement

**Figure 6:**
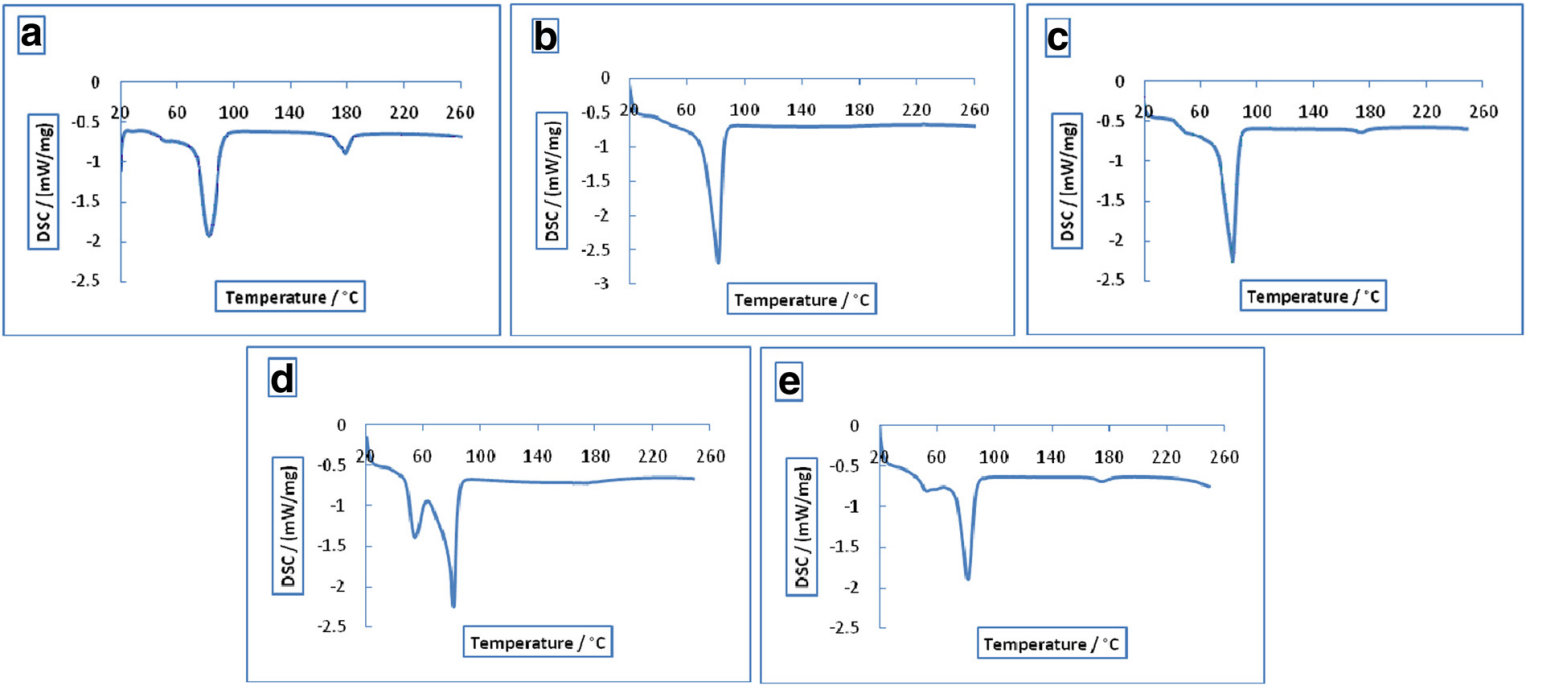
DSC thermograms PLGA, and α1AT -loaded PLGA nanoparticles: (a) PLGA 50:50; (b) PLGA 75:25; (c) α1AT -loaded nanoparticles prepared by nonaqueous emulsion method; (d) α1AT -loaded nanoparticles prepared by double emulsion method.

The DSC curves for PLGA 50:50, PLGA 75:25 and α1AT- loaded PLGA nanoparticles prepared using the nonaqueous and double emulsion method were detected and shown in Figure 6. α1AT exothermal peak were not present in α1AT-loaded nanoparticles which indicate that no crystalline α1AT was found in nanoparticles. The similar melting transition properties of loaded and unloaded nanoparticles show the PLGA polymer remained unaffected during encapsulation. The DSC thermograms of pure PLGA materials and α1AT-loaded nanoparticles were shown in Figure 6.

### Evaluation of Cytotoxicity

**Figure 7:**
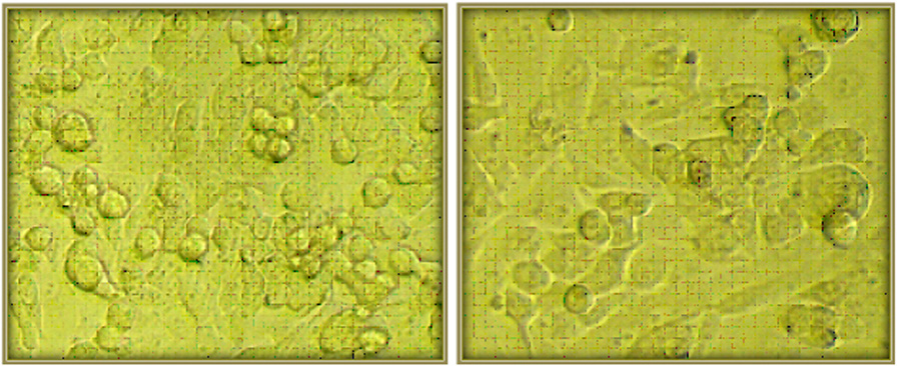
Cor L105 lung epithelial-like cells before and after treatment with nanoparticles.

In this study, cell cytotoxicity of α1AT- loaded nanoparticles was evaluated using Cor L105 lung epithelial-like cells. The results show that the viability of cells treated with different concentrations of free and loaded nanoparticles remains unchanged and cells retained more than 80% of their viability. Furthermore, the morphology of cells before and after treatment with nanoparticles was similar (Figure 7).

## Discussion

α1AT inhibits a broad range of proteases and protects the lung from neutrophil elastase during inflammation or infection [1,2]. This inhibitor is an acute phase protein the plasma concentration of which increases manyfold upon inflammation [15]. The absence or inefficient function of α1AT in the lungs leads to uncontrolled function of elastase and elastin breakdown, resulting in respiratory problems such as COPD and emphysema [3-5]. Association between α1AT and a number of diseases including asthma, rheumatoid arthritis, anterior uveitis and systemic lupus erythematosus suggests that α1AT is not only an anti-inflammatory protein but also an immune system regulator [7-9]. α1AT regulates lymphocyte proliferation and cytotoxicity, mediates monocyte and neutrophil functions. Besides, researchers have shown that the protease-antiprotease imbalance is an important factor in the pathogenesis of COPD and other pulmonary diseases, such as bronchitis. COPD is one of the most important causes of irreversible lung damage and thus the fourth most common cause of death in the U.S. In this process, exogenous proteolytic enzymes lead to lung damage. Besides different physiological roles of α1AT including the control of insulin secretion, antiprotease activity, protecting β-cells against cytokine-induced apoptosis, acting as an anti-inflammation compound [14-16], it is also regarded as an antiapoptotic factor in lung epithelial cells [9]. A recent study has shown that α1AT can slow down the loss of insulin producing cells in diabetic patients who are at the early stages of disease. This study which is in phase II of clinical trials demonstrates the effect of α1AT in preservation of β-cells' function [17,18]. Therefore, only with appropriate and adequate concentrations of α1AT the lungs' correct function can be maintained. One of the treatment strategies for optimum activity of α1AT during inflammation is replacement therapy using intravenous infusion (Prolastin, Zemaira, Aralast and Glassia) [1,6]. In the infusion form, only 10%-15% of α1AT reaches the target organ. Another possible treatment strategy is through airway delivery. In this form of treatment not only the aerosolized α1AT directly reaches the target organ, but also prevents the accumulation of excess drug in the blood, therefore, a much lower level of drug is required [25,26]. Also immune reactions arising from the retention of drug in the peripheral circulation are avoided.

Besides the different forms of α1AT which are all plasma-derived products and have disadvantages, such as viral contaminations and high costs, the recombinant forms of α1AT derived from eukaryotic hosts such as yeast, are currently under study as a therapy for α1AT deficiency [35], however, these are not yet commercially available. Furthermore, in addition to the development and optimization of recombinant forms of α1AT, efforts on packaging α1AT in micro- and nanoparticles for pulmonary delivery are also under investigations. The lung tissue has been mainly used as a delivery route for many drugs because of its unique anatomical and physiological properties. With the development of new inhaler devices, the role of the lung tissue in drug delivery becomes more important and dominant. In addition to chemical drugs, especially for the treatment of pulmonary diseases, delivery of macromolecules such as different antibodies and hormones are also part of the research of many pharmaceutical companies [26].

Various antibodies, and insulin, which are now objectives at different phases of clinical trials, are examples of such medicines.

The pulmonary drug delivery strategies for protein and peptide-based medicines are based on using particles with a lipid origin (liposomes) and that are polymeric (PLGA, Chitosan) [20,24].

Besides the type of particle, physicochemical properties such as surface charge, size and stability are important factors in the selection of particles for lung drug delivery [34]. Among particles, PLGA is approved by the FDA for controlled and sustained drug delivery because it is a biocompatible, bioavailable and stable polymer that is not toxic to cells [20-31]. Various peptides and chemical drugs are delivered with PLGA, such as rifampicin, isoniazid, pyrazinamide, nuclear factor κB decoy oligodeoxynucleotide [36].

Like oral drug delivery, for pulmonary delivery the carrier must be biocompatible, small to pass through the intestinal barrier (M cell), have size dispersity, physical stability and higher encapsulation efficiency [37,38].

α1AT involves being internalized by endothelial cells mostly by the process of clathrin-mediated endocytosis. In the case of particle uptake, clathrin-coated pits expand and enclose particles of around 200 nm [38]. In addition, particles with an aerodynamic diameter of less than 0.1μm are able to reach the deepest regions of the lung, the alveoli [39].

The emulsification solvent evaporation technique is another common process for the synthesis of PLGA nanoparticles because of its simplicity and high encapsulation efficiency. The single emulsion method is only suitable for hydrophobic drugs and is not efficient for protein or peptide drugs. The oil-in-oil (o/o) emulsification technique which is known as a nonaqueous emulsion method is a new and efficient method for encapsulation of hydrophilic drugs. The double emulsion method is also known as a suitable procedure for encapsulating hydrophilic drugs with high efficiency [20,33,34]. Particle size plays an important role in lung deposition, along with particle velocity and settling time. As particle size increases above 3 μm, there is a shift in aerosol deposition from the periphery to the conducting airways. Oropharyngeal deposition also increases as particle sizes increase above 6 μm. Exhaled loss is high with very small particles of 1 μm or less. These data support the view that particle sizes of 1-5 μm are best for reaching the lung periphery, while 5-10 μm particles deposit preferentially in the conducting airways.

Aerosol devices in clinical use produce heterodisperse (also termed polydisperse*)* particle sizes, meaning that there is a mix of sizes in the aerosol. This is contrasted with monodisperse aerosols aerosols, which consist of a single particle size [38,40].

Polydispersity may have an advantage by increasing the probability that at least one fraction of the aerosol will reach the desired region of the lung**.**

As the imbalance of proteases and anti-proteases in the lungs is an important factor in the pathogenesis of COPD and other pulmonary diseases [12,13,16], hence, the main candidate for pulmonary delivery is the main lung anti-protease, α1AT. The necessity of the anti-protease presence in the lung for controlling the increase in activity of elastase during inflammation is important. Therefore in order to obtain such results, drug delivery strategy must be based on the type of particle (lipid or polymer), particle size, dispersity, stability, efficiency and efficient drug release at appropriate times. For the best result, anti-protease must be appropriately packaged to be released in sufficient amounts in different parts of the lungs in COPD patients. The released drug must perform its anti-protease activity locally and be absorbed efficiently by the lung epithelial membrane to raise appropriate concentrations of the anti-protease in the interstitial space (the main target place of protease) and blood for further support.

In this project, the evaluation of α1AT release and particle properties for the purpose of α1AT aerosolization was carried out. We used PLGA because drug release can be controlled by its molecular weight and the ratio of lactide to glycolide used for the polymerization. PLGA has been approved as a safe polymer because it undergoes hydrolysis to form lactic and glycolic acids. Lactic acid is degraded to carbon dioxide and water through citric acid and glycolic acid is excreted with urine or oxidized to glyoxylic acid [20,][25-28].

The results of this study showed that the release profiles for PLGA at the 50:50 and 75:25 ratios were different. By using PLGA at the 50:50 ratios, fast degradability kinetics was obtained. The initial burst was higher with PLGA 50:50 but the slow release period was more extended when using PLGA at the 75:25 ratios. The induction period and final release period are quite similar in both polymers. In the other words, using these two ratios of polymeric monomers together sufficient release of anti-protease for efficient function will be obtained [25,26,30,31].

**Figure 8:**
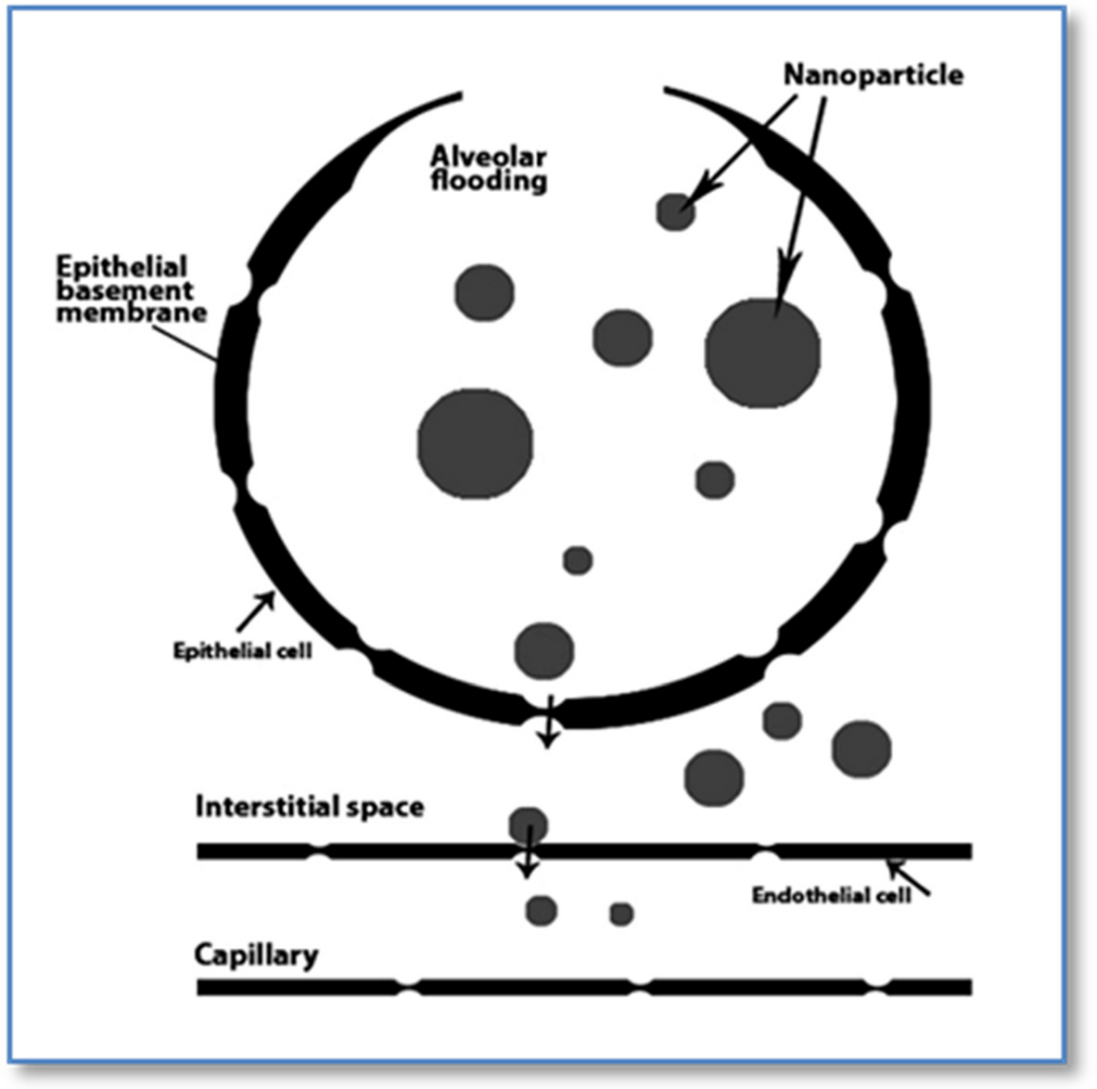
a schematic representation of nanoparticle passage and decomposition in lung.

Meanwhile, the amount of drug to PLGA of 5/25 mg was selected to obtain the desirable loading efficiency, mean particle size and distribution and drug release profile. The suitable particle size varies based on the target organ. For example for oral recombinant drug delivery which needs particle and loaded protein-based drug absorption through the intestine, particles' diameter must not exceed 10 μ because larger particles cannot be absorbed by the M-cell containing Peyer's patch in the intestine via endocytosis. In the lungs, different endocytosis and phagocytosis mechanisms also exist based on the cells' distribution. Particle sizes ranging from 0.5 to 2 μ are phagocytized by lung macrophages. Furthermore, ultrafine particles may be endocytized by lung endothelial cells, which then enter blood circulation. The local neutrophil elastase inhibitory effect of α1AT is mainly in the interstitial and alveolar spaces, with the entrance to blood circulation being less important. Therefore, particle sizing for aerosol purposes is very important in the distribution, delivery and release of the carried drug. α1AT delivery requires accessibility of drug to the lower and middle respiratory tracts which need the endocytosis process. Therefore the particles' dispersity with a size range of 100 to 1000 nm is very suitable for drug delivery. As a result, the preparation method of nanoparticles must account for the size polydispersity (Figure 8).

The results obtained in this study showed a mean particle size of 300 nm, and as mentioned previously, in this heterogenic mixture of particles, most particles had a diameter less than 500 nm which is an appropriate size for endocytosis by the lung endothelial cells. The endocytosis process causes the entrance and release of α1AT into the interstitial spaces where neutrophil elastase secretion affects elastin, therefore, leading to a targeted treatment. On the other hand, particles with more than 500 nm in diameter reside in the alveolar region and the released drug inhibits the elastase secreted by neutrophils in this region.

In addition to the location of release, the amount of drug to be released must be in proportion to the severity of inflammations at a specific period of time. The threshold concentration of AAT in circulation is 11 μM, therefore, we can assume this level as a protective concentration for the lungs against protease invasions during inflammation. For this purpose, the amount of drug release for obtaining the threshold concentration at a specific period of time must be in a range that does not impair the function of functional lung cells, such as the alveolar epithelial and endothelial cells. One of the possible problems is the amount of particles carrying this concentration of drug. For the investigation of this matter, we assume that if the drug release efficiency was only %10, therefore to investigate the toxic effect, the tenfold higher amount of loaded PLGA was applied to ensure the concentration of 11 μM. Cor L105 cells were treated with different concentrations of loaded nanoparticles and results show no toxicity toward the cells.

## Conclusions

In summary, a protein loaded-PLGA nanoparticles was synthesized. In synthesis procedure (2-hydroxypropyl)-β-cyclodextrin and threhalose were employed which decrease loss of protein activity and act as cryoprotactant, respectively. Nanoparticles have a wide range of particle size which can be deposit in different parts of the respiratory system especially in the deep lung. Various lactide to glycolide ratio of the copolymer show**s** different release profile of the drug which can significantly covers extended and rapid drug release in one formulation. Nanosized particles enable the drug to reach deep into the lungs for maximum benefit. This mix formulation shows a great potential for protein drug delivery in pulmonary diseases especially in obstructive lung disease for rapid, direct and extended delivery of drug allowing high local drug concentrations.

## Material and methods

### Materials

Poly (D, L-lactide-co-glycolide) (PLGA), with a copolymer ratio of lactide: glycolide 50:50 (Mw 40,000-75,000) and 75:25 (Mw 66,000-107,000) were purchased from Sigma Aldrich (USA). Poly (vinyl alcohol) (PVA) with a molecular weight of 89,000-98,000 and Hydroxypropyl-β-cyclodextrin (HP-β-CD) were obtained from Sigma Aldrich (USA).

Alpha one-antitrypsin from human plasma and 3-(4, 5-dimethylthiazol-2-yl)-2, 5-diphenyltetrazolium bromide (MTT) were bought from Sigma-Aldrich (St. Louis, MO, USA). RPMI 1640, fetal bovine serum (FBS), glutamine and penicillin/streptomycin were obtained from Gibco BRL (now part of Invitrogen Corporation, Carlsbad, CA, USA).

Methylene chloride, acetone, Span80, n-hexane, viscous Paraffin, Acetonitrile and DMSO were purchased from Merck (Germany). All reagents were of analytical grade.

### Methods of preparation

**Figure 9:**
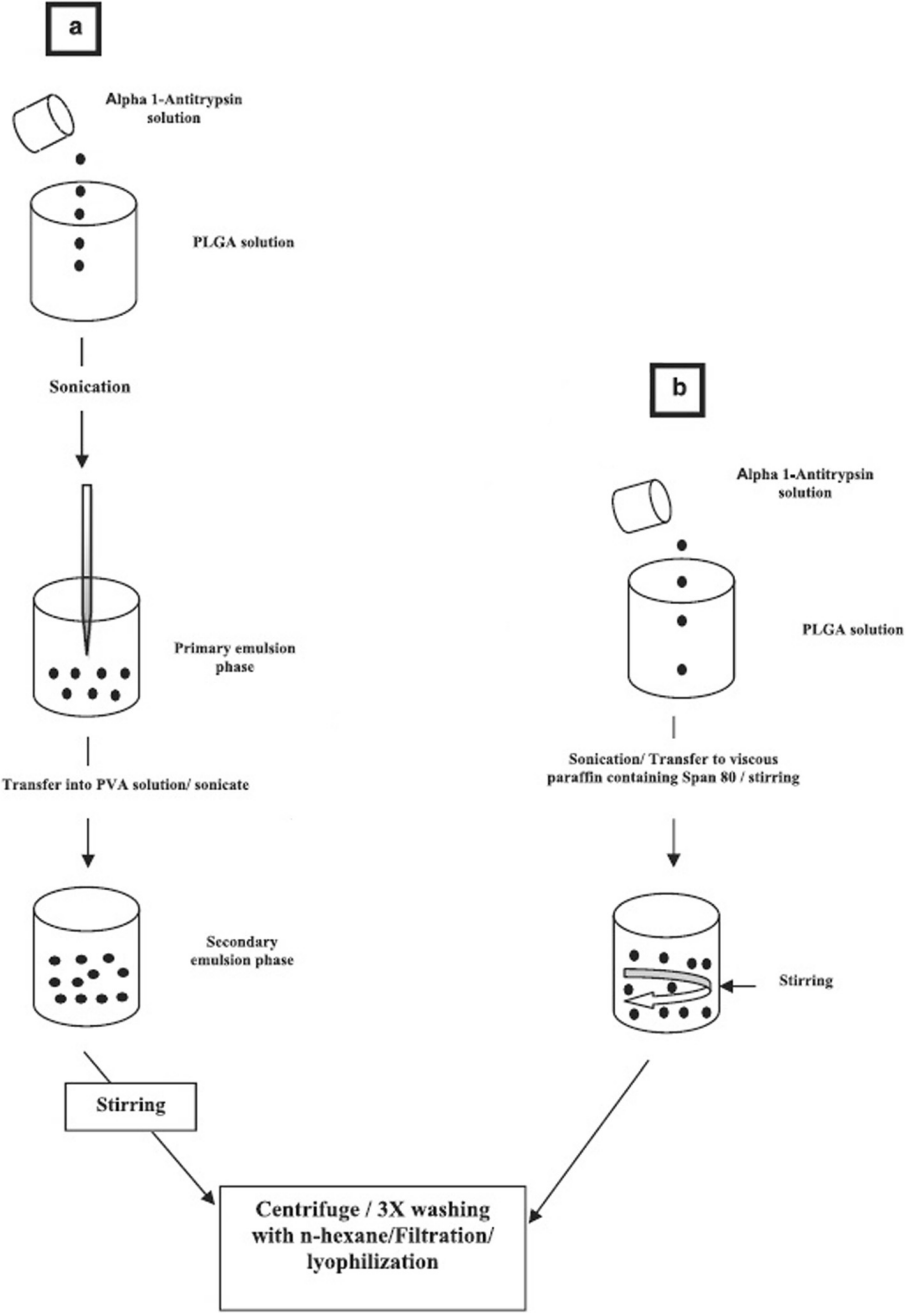
Two different methods of nanoparticle preparation by a) double emulsion and b) nonaqueous emulsion technique.

In this study Alpha one-antitrypsin (α1AT) loaded particles were prepared by two different techniques (Figure 9):

#### Nonaqueous emulsion technique

Briefly, this technique is carried out by dissolving 25mg of PLGA (50:50 and 75:25) in 3ml of acetonitrile. The solution was stirred for 30 min. 5mg of α1AT was dissolved in double distilled water and was added drop-wise to the abovementioned solution. The mixture was poured drop- wise into 50ml of high viscosity paraffin containing 400 μl of %1 v/v Span80 and the resulting mixture was stirred 500rpm for 4 hours to at room temperature using a magnetic stirrer to ensure solvent evaporation and nanoparticle hardening. Nanoparticles were collected by centrifuge at 20000 rpm for 45 min, washed twice with n-hexane to remove mineral oil, lyophilized (LaboGene ScanVac CoolSafe freeze dryer) using 2.5% trehalose dihydrate as cryoprotectant and stored at -20° C [41].

#### Double emulsion technique

In this method 5 mg of protein was dissolved in 0.5 ml of citrate buffer (pH=8) containing 0.5 mg/ml BSA solution and 5% (2-hydroxypropyl)-β-cyclodextrin, which was previously reported to decrease loss of protein activity in interior water phase [42]. The protein solution was emulsified with 3ml of 3:1 methylene chloride: aceton containing 25 mg of PLGA (50:50). Emulsification was carried out by ultrasonicator (Elmasonic S 60 (H), Elma, Elma Hans Schmidbauer GmbH & Co. KG, Germany) at amplitude of 5 for 20 seconds in an ice-cooled bath. The w/o primary emulsion was added to 10 ml of 2% (w/v) aqueous PVA in a drop-wise manner and then emulsified with high speed hemogenizer for 2 min at 10000 rpm. The w/o/w double emulsion was added to 80ml double distilled water and was stirred in 500 rpm for 2 hours to allow the solvent evaporation. The hardened nanoparticles were collected by centrifuge at 20000 rpm for 45 min and washed thrice with sterile deionized water. Finally particles were freeze-dried using 2.5% trehalose dihydrate as cryoprotectant.

## α1AT –loaded nanoparticle characterization

### Scanning electron microscopy observation

Scanning electron microscopy (SEM) was performed using TESCAN Vega LMU (USA) for morphology examination. One drop of concentrated 2 mg/ml aqueous suspension samples were deposited on aluminum stubs and dried in a desiccator at room temperature to obtain a uniform layer of nanoparticles. Samples were coated with a 15 nm of gold layer using EMITECH K450X sputter-coater (England). Coating was done at 20 mA for 2 min.

### Determination of particle size distribution

The laser dynamic light scattering (DLS) technique was used for the determination of particle mean diameter and distribution. Samples were prepared by suspending 5mg of each sample in 5 ml of sterile deionized water and sonicated at amplitude of 40 and 0.5-sec pulse cycle for 5 min (Hielscher ultrasonics GmbH, Germany). Measurements were carried out using SEMATECH light scattering (France) with a He-Ne laser (λ = 633) at room temperature.

### X-Ray diffraction (XRD) measurement

X-ray powder diffraction patterns were measured using a Siemens FK 60-04 diffractometer (France). Measurements were carried out with Fe-Kα radiation at 35 Kv and 25 mA. Film samples were scanned continuously over an angular range between 4° and 90° (2θ) with a step size of 0.02°. The scan step time was 1.2°/min.

### Encapsulation efficiency

Encapsulation efficiency was determined by hydrolyzing. 5 mg of prepared nanoparticle was dissolved in 0.5 ml of sodium hydroxide 1 M and incubated at 37°C for 14h. The solution was neutralized by adding 0.5ml of hydrochloric acid 1M. The solution was centrifuged for 5 min at 13000 rpm and the supernatant was analyzed for α1AT content. Protein concentration is determined by measuring intrinsic fluorescence using fluorescence spectrophotometer (Varian Cary Eclipse, Australia). The fluorescence intensity of the samples is measured from a standard curve ( Additional file 1: Table S1). A series of dilutions ranging from 2.5 to 200 μg/ml of α1AT were made using phosphate buffer saline (PBS), pH 7.4 as diluent.

Encapsulation efficiency was determined by subtracting the total amount of protein added during synthesis from the concentration of protein obtained through standard curve.

### In vitro release profile

For drug release studies 10mg of lyophilized A1AT loaded PLGA obtained from different techniques were suspended in 1ml of PBS pH 7.4 in a 2 ml eppendrof tube by sonication at amplitude of 40 and 0.5-sec pulse cycle for 2 min. Tubes were placed in shaker water bath (Memmert, Germany) with constant shaking of 100 strokes per minute in 37°C. At different time intervals, samples were centrifuged (Sigma microfuge, Germany) at 13000 rpm for 20 min; aliquots of 500 μl were withdrawn and replaced with the same volume of fresh PBS. Particles were re-dispersing each time. The amount of released α1AT was determined by measuring intrinsic fluorescence mentioned above.

### Protein polymer interaction study

The Fourier transformed infrared (FTIR) spectroscopy of α1AT, PLGA and α1AT-loaded PLGA particles were carried out with Bruker Tensor 27 (Bruker Optik, Germany) in order to evaluate the kind of possible chemical interaction occurring between protein and polymer. The absorption peaks in infrared spectrum show the frequencies of vibrations between bonds of atoms constituting the material. For sample preparation few drops of pure PLGA (50:50 and 75:25) in acetonitrile and pure α1AT were placed onto KBr pellets and the absorbance was scanned over the range of 3600-600 cm^­1^ at a resolution of 4 cm^1^ at room temperature. Lyophilized α1AT-loaded PLGA particles were ground into powder and mixed with IR grade KBr to produce pellet. The background was obtained against pure KBr pellet.

### Differential scanning Calorimetry measurement

Differential Scanning Calorimetry (DSC) was carried out by NETZSCH DSC 200 F3 Maia, Germany to investigate the thermal property of protein inside the particles. 5 mg of α1AT, PLGA and α1AT-loaded PLGA particles were weighted and loaded onto a standard aluminum pans (2mm high & 4mm diameter). Samples were cooled to -80°C and heated from 25°C to 250°C with a heat flow rate of 10°C/min. Calibration was carried out by an aluminum pan as a reference.

### Cell culture

Human lung epithelial-like cells, Cor L105 (cell collection of Pasteur Institute, Iran) were cultivated in RPMI 1640 media supplemented with 10% heat-inactivated FBS and 2 mM L-glutamine , NaHCO_3_ and 100 units/mL penicillin, 100 mg/mL streptomycin at 37° in O_2_ 5%/CO_2_ 5% and 95% humidity. The medium was replenished and the cells were subcultured by trypsinization.

### Evaluation of Cytotoxicity

To determine the cytotoxic effect of loaded nanoparticles on cell growth, 3-(4,5- dimethylthiazol-2-yl)-2,5-diphenyltetrazolium bromide (MTT) cell viability assay, which assesses the mitochondrial activity in living cells, was carried out. Cor-L105 human lung epithelial–like cells were plated at a density of 4 × 10^4^ cells/well on a 96-well plate for 24 hours in a humidified incubator at 37°C and %5 CO_2_. Then, cells were treated with different nanoparticle concentration of 0 to 1.5 mg/ml for 24 hours. Selected concentrations correspond to protective threshold of α1AT which is 11μM or 0.8 mg/ml. Cells which were incubated only in culture medium were used as control. After 24 hours, cells were washed with fresh PBS and inhibition in cell growth was assessed by adding 25μl of the MTT solution (5 mg/mL in PBS) to each well. Absorbance was measured at 540 nm with microplate reader.

## Competing interests

The authors declare that they have no competing interests.

## Authors’ contributions

Dr. SH was main supervisor of this research in Tarbiat Modares University and National Institute of Genetic Engineering and Biotechnology. Dr. NP wrote this manuscript and carried out most of the experiments. Professor ASL from Tarbiat Modares University and National Institute of Genetic Engineering and Biotechnology analyzed the data and advised on experimental part. Professor MG from Baqiyatallah University of Medical Sciences contributed gave us valuable guidance to improve this work especially in lung pathology. All authors read and approved the final manuscript. All authors read and approved the final manuscript.

## Supplementary Material

Additional file 1: Table S1 Standard curve for determination of protein concentration Click here for file
