# Supplementary material for: Encapsulation of Alpha-1 antitrypsin in PLGA nanoparticles: In Vitro characterization as an effective aerosol formulation in pulmonary diseases
Source: J Nanobiotechnology. 2012 May 20;10:20. doi: 10.1186/1477-3155-10-20 (PMC3485170; doi:10.1186/1477-3155-10-20)
Supplement: Additional file 1: Table S1 — Standard curve for determination of protein concentration [file 1477-3155-10-20-S1.docx]

Table 1: Standard curve for determination of protein concentration

| Standard | Concentration | Reading |
| --- | --- | --- |
| Std 1 | 2.5 | 2.576 |
| Std 2 | 10.0 | 5.349 |
| Std 3 | 25.0 | 9.176 |
| Std 4 | 50.0 | 32.433 |
| Std 5 | 100.0 | 48.268 |
| Std 6 | 150.0 | 81.552 |
| Std 7 | 200.0 | 96.435 |
